# Case report: The first known case of male retroperitoneal mesonephric-like adenocarcinoma

**DOI:** 10.3389/fonc.2024.1433563

**Published:** 2024-10-28

**Authors:** Baohong Hu, Ying Liu, Jingjing Tang, Ping Yang, Di Sun

**Affiliations:** ^1^ Department of Oncology, Yantai Yuhuangding Hospital of Qingdao University, Yantai, China; ^2^ Department of Gynecology, Haiyang Maternal and Child Health Hospital, Yantai, China; ^3^ Department of Pathology, Yantai Yuhuangding Hospital of Qingdao University, Yantai, China

**Keywords:** male, retroperitoneum, MLA, pathological characteristics, literature review

## Abstract

**Aim:**

We aimed to analyze the clinico-pathological and molecular features of mesonephric-like adenocarcinoma (MLA) to enhance understanding of this tumor type.

**Methods:**

This is the first case of MLA occurring in the retroperitoneum of a male patient. Clinico-pathological and molecular characteristics were analyzed, and the relevant literature was reviewed.

**Results:**

A 65-year-old elderly male was admitted to the hospital with mild bilateral dull pain in the lumbar region for more than 1 month, accompanied by a feeling of dysuria. CT tomography revealed a retroperitoneal tumor. While tumor immuno-histochemistry was positive for CK, CK7, Vimentin, PAX-8, CD10, GATA-3, EMA, and CR to varying degrees, it was negative for P53, WT-1, HMB45, MelanA, CD117, DOG-1, CD34, S-100, ER, PR, AR, CEA, α-inhibin and TTF-1. Ki67 index was <10% in most areas and was approximately 30% in the hotspot areas in the glandular ductal region. Molecular detection (Next-generation sequencing method, 425-gene panel from NanjingShihe Gene Biotechnology Co., Ltd. for targeted DNA enrichment): No clinically significant variants detected. The final pathological diagnosis was a retroperitoneal malignant tumor consistent with a well-moderately differentiated MLA.

**Conclusion:**

MLA in the retroperitoneum of men has not been reported yet. The diverse morphology and unclear molecular characteristics of this tumor mandate careful diagnosis for good clinical outcomes.

## 1 Introduction

Mesonephric-like adenocarcinoma (MLA) is a newly defined group that encompasses specific types of adenocarcinomas of the ovary and uterine corpus (1), which has been included in the 2020 World Health Organization (WHO) classification of tumors of the female genitalia (2). Studies have shown that these tumors predominantly occur in females, and no reports of cases in males exist. We report a case of male retroperitoneal MLA, and present clinico-pathological features and molecular characterization in the patient in an attempt to enhance understanding of this tumor, in conjunction with an extensive literature review.

## 2 Case description

### 2.1 Patient details and initial diagnosis

Informed consent has been obtained from the participant and the study was approved by Ethics Committee of Yantai Yuhuangding Hospital. The patient was a 65 years old male, with bilateral lumbar pain for more than one month. The pain was described as mild and dull, and was accompanied by a feeling of incomplete urination. The patient had no history of hematuria, increased urinary frequency, urgency, urinary pain, fever, or fatigue. The patient was admitted to an outside hospital and underwent urological ultrasound, which showed 1:abnormal development of both kidneys, hydronephrosis with multiple stones in the left kidney. 2: Multiple cystic structures below the right kidney and peripheral solid nodules. 3: Enlarged prostate with calcified foci, which was not treated, and the patient came to our hospital for further treatment. His routine blood tests were as follows: WBC: 8.09X10/L, lymphocytes: 10.9%, and neutrophils: 6.59 X10/L. The tumor indices were: glycogen antigen-125:6.48 U/ml, carcinoembryonic antigen: 2.24 ng/ml, prostate-specific antigen: 8.80 ng/ml, and squamous cell carcinoma-related antigen: 0.6 ng/ml.

**Figure 1:**
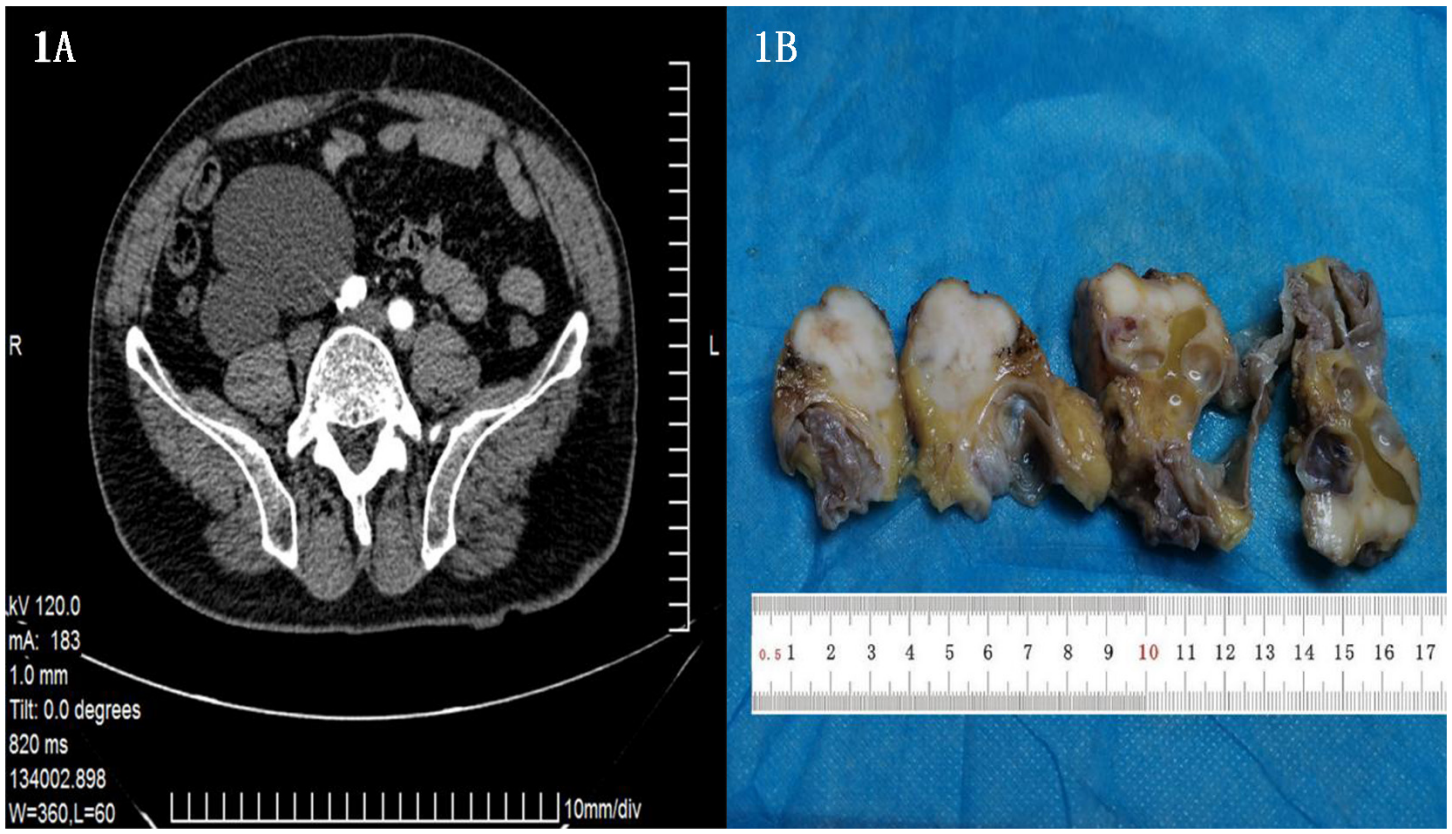
**(A)** Enhanced CT showing a cystic solid space occupying lesion under the right kidney, with a clear boundary measuring approximately 8.1 × 7.7 × 12.2 cm. **(B)** A single cystic solid tumor measuring 7*6*4 cm was evident in the retroperitoneum. The cystic area had a diameter of 3 cm, with a smooth inner wall of 0.1 cm thickness. The solid area had diameter of 4 cm, and a cut section was found to be gray white in color, hard, and possessed an intact capsule.

Contrast-enhanced CT revealed a cystic solid occupancy below the right kidney with clear borders. The mass was located approximately in front of the right psoas major muscle, medial right common iliac artery, and lateral of the ascending colon. The tumor measured approximately 8.1×7.7×12.2 cm. While no obvious enhancements were evident in the arterial phase of the enhanced scan and in the cystic portion, a slight enhancement was observed in the solid part in the venous and delayed phases. (**Figure 1A**, arrow indicates tumor).

### 2.2 Pathological and molecular characterization

The excised tumor was a single cystic-solid mass measuring 7*6*4 cm, with the cystic area having a diameter of 3 cm, and a smooth inner wall of 0.1 cm thickness. The solid area had diameter of 4 cm, with a grayish-white and hard cut surface and an intact peritoneum (**Figure 1B**).

**Figure 2:**
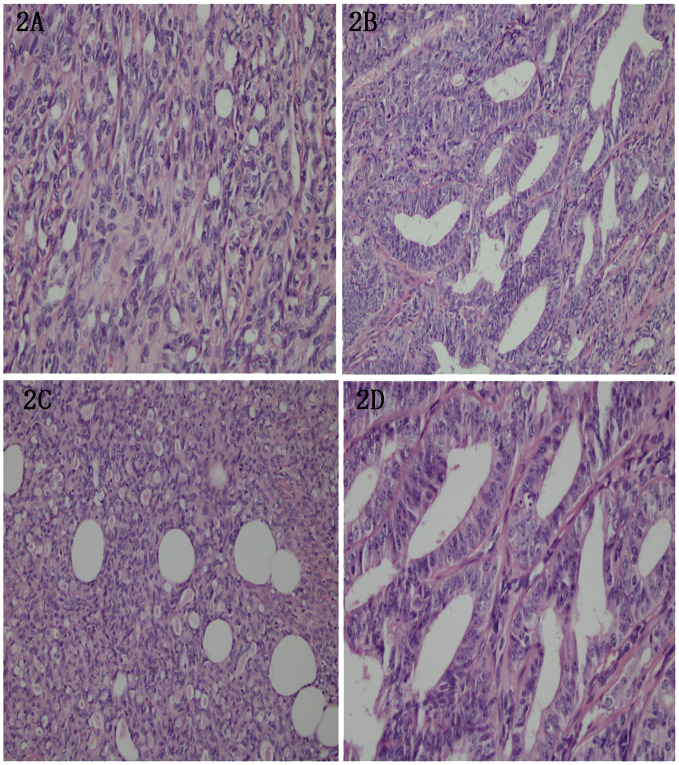
**(A)** Tumor cells showing bidirectional differentiation, of which some were spindle shaped. HE, (X200). **(B)** Tumor cells showing glandular tubular and tubular growth. HE, (X100). **(C)** The tubule epithelium was cuboidal or flat. HE, (X100). **(D)** Pink matter and few mitotic events with a frequency of 2-10/HPS in the cavity. Nerve invasion was evident, along with interstitial fibrous tissue hyperplasia with hyaline degeneration. HE, (X100).

The tumor demonstrated a diverse patho-histological patterns, with spindle-shaped, glandular, and tubular growths. The tubular epithelium was cuboidal or flattened, and eosinophilic material deposition was observed. Additionally, few nuclear divisions were visible with a frequency of 2-10/HPF. Neural invasion was evident, and interstitial fibrotic tissue proliferation was accompanied by hyaline degeneration (**Figure 2**). The majority of the tumor areas display low-grade morphology, with focal regions showing intermediate-grade features.

**Figure 3:**
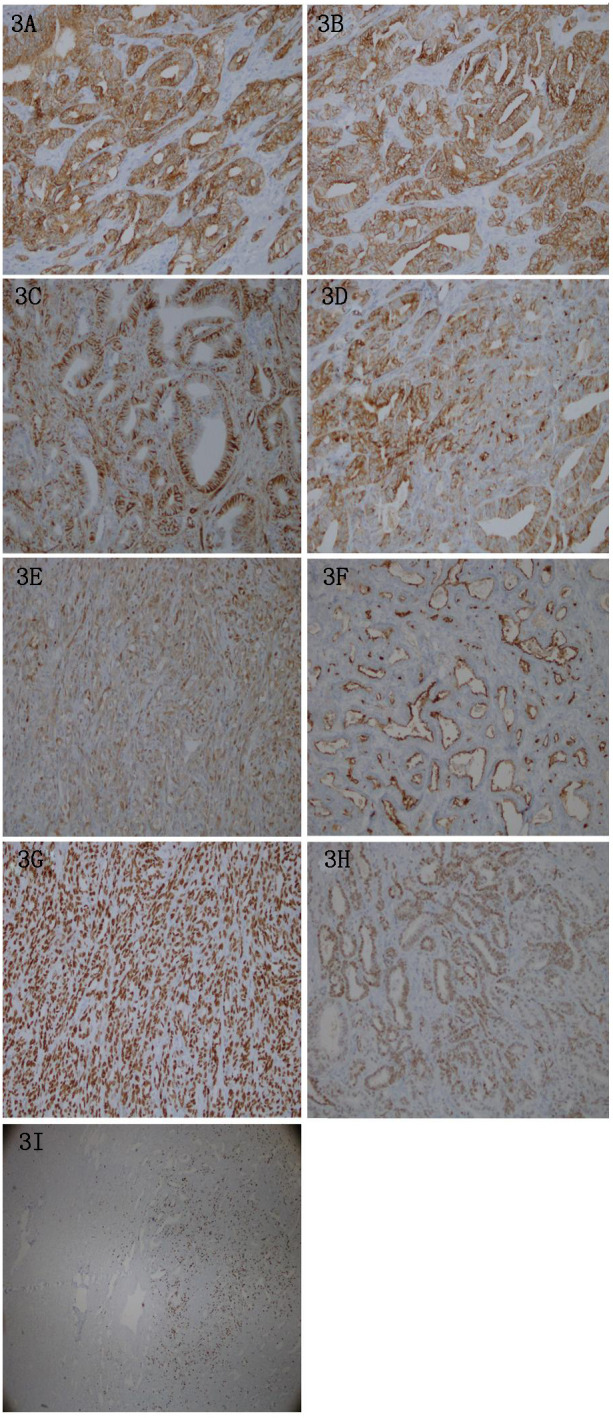
**(A)** Tumor cells were diffusely positive for CK, EnVision method. (X200). **(B)** Tumor cells were positive for CK7, and part of adenoid structure was weakly positive. EnVision method. (X200). **(C)** Tumor cells and the glandular tube were positive for Vimentin, and the rest was weakly positive. EnVision method. (X200). **(D)** Tumor cells were positive for EMA, mainly lumen margin positive, mostly positive. EnVision method. (X200). **(E)** Tumor cells were partially positive for CR. EnVision method. (X200). **(F)** Tumor cells were positive for CD10 at the lumen margin. EnVision method. (X200). **(G)** Tumor cells of the glandular duct were weakly positive for Pax-8, and the rest were diffusely strongly positive. EnVision method. (X200). **(H)** Tumor cells were GATA-3 foci positive. EnVision method. (X200). **(I)** The Ki-67 proliferation index in most areas of tumor cells was less than 10%, and about 30% in the hot spots in glandular areas. EnVision method. (X200).

Immuno-histochemical analysis revealed the following results: CK (diffuse +), CK7 (+, with some adenotubular-like structures weakly +), Vimentin (+, adenotubular +, and few remaining weakly +), PAX-8 (adenotubular weakly +, and the rest diffusely strongly +), CD10 (luminal margins +), GATA-3 (foci +), EMA (+, luminal margins predominantly +, and most +), CR (some +); P53 (-, nonsense mutation); PMS2(+), MLH1(+), MSH2(+), MSH6(+); WT- 1 (–), HMB45 (–), MelanA (–), CD117 (–), DOG-1 (–), CD34 (–), S-100 (–), ER (–), PR (–), AR (–), CEA (–), inhibin (–), TTF-1 (–), and Ki67 positivity of <10% in most regions, and of approximately 30% within hotspots in glandular ductal regions. (**Figure 3**).

Molecular detection (Next-generation sequencing method): No clinically significant variants detected.

Based on the morphology, surgical, and immuno-histochemical findings, a final pathological diagnosis of a retroperitoneal malignant tumor consistent with a well-moderately differentiated MLA was made.

## 3 Diagnostic assessment

Based on the imaging findings, a preoperative diagnosis of retroperitoneal mass (right infrarenal) was made. After clinical improvement of relevant examinations, laparoscopic resection of the retroperitoneal mass was performed. The mass was located in the posterior aspect of the colonic appendix with intact peritoneum, which was completely removed surgically. The patient had suffered from allergic asthma for 15 years, and denied any history of surgical trauma, family genetics or related diseases.

The patient did not undergo any post-operative treatment, such as, radiotherapy or chemotherapy. He has remained tumor-free on follow up for 21 months until now.

## 4 Discussion

The mesonephric and Mullerian ducts travel in parallel during the embryonic period, and form the epididymis, vas deferens, seminal vesicles, and a portion of the prostate and urethra in males. The mesonephric ducts undergo degeneration in females due to lack of testosterone (1–7), and adults only carry remnants of the nonfunctional mesonephric ducts that are usually located in the median ovary, broad ligament, or lateral wall of the cervix, and rarely in the vagina or body of the uterus with low probability of malignancy. Mesonephric adenocarcinoma (MA) is thus a rare malignancy of the female genital tract, usually located in the cervix and vagina that originates from the embryonic remnants of the mesonephric tubules and ducts, and accounts for less than 1% of all gynecologic malignancies (8). Within this context, MA of the female upper genital tract is referred to as mesonephric-like adenocarcinoma (MLA), on account of unproven association with mesonephric remnants (2, 9). Reports have suggested that mesonephric-like adenocarcinoma (MLA) originates from mullerian ducts, and is not associated with mesonephric remnants (10). While cases of MLA have been documented in females, no reports on male patients exist. Literature review revealed that mesonephric ductal remnants may be located not only in the female reproductive system, but also in the retroperitoneum, adjacent to the kidneys, posterior to the colon, and near the head or tail of the pancreas (11, 12).

The present study is the first report of a retroperitoneal MLA in a 65-year-old elderly male who presented at the clinic with mild bilateral lumbar dullness and pain that was accompanied by dysuria. Imaging revealed a cystic-solid retroperitoneal occupancy with clear borders, which was consistent with the findings of Koh et al., who reported that MLA manifests as either mixed solid and cystic masses, or pure solid masses in imaging studies (9), with the typical presentation often being suggestive of an MLA tumor. MLAs exhibit a variety of patho-histological patterns (13), including tubular, adenotubular, papillary, reticular, solid, glomerulonephric, gonadotubular/trabecular, sieve, and osteosarcomatous growths, with the possibility of combinations of these patterns within a single tumor. The common spindle-like osteosarcoma-like differentiation pattern includes tubular and glandular tubular growths. The tubules usually comprise small round tubules that grow in a back-to-back or diffusely infiltrating pattern with cuboidal or flattened epithelium and eosinophilic material deposition was observed. Although adenoductal structures consist of large glands or papillary-like formations, the tumor cells generally have mild to moderate cytological atypia, which was also consistent with the pathological observations made in the present case. The tumor foci in two of the ovarian MLA tumors reported by Koh et. al, could be interpreted as severe cytokinetic polymorphisms with scattered areas of extensive coagulative tumor necrosis (9). The 2-10/HPF nuclear schizophrenic pixels observed in the present case were slightly lower tumor than the numbers reported in literature ranging within 3-50/HPF. While mesonephric ductal hyperplasia is often present in the periphery of most tumors with peripheral nerve invasion (14), it was not evident in our case despite the presence of peripheral nerve invasion.

The MLA immune-phenotypes that have been reported in the literature (9, 13, 15) are predominantly positive for PAX-8, CD10 (luminal rim expression), TTF1, and/or GATA3, with reverse reciprocal staining patterns for GATA-3 and TTF-1. Additionally, these tumors displaying non-diffuse P16 immuno-reactivity and wild-type P53 immuno-staining patterns, with negative results for WT-1, ER, PR, AR, CEA, and inhibin along with a ki-67 positivity index of 50% (15–17). Immuno-histochemical analysis revealed that while CK, CK7, Vimentin, PAX-8, CD10 and GATA-3, EMA, CR were positively expressed to varying degrees; P53(-, nonsense mutation); WT-1, HMB45, MelanA, CD117, DOG-1, CD34, S-100, ER, PR, AR, CEA, inhibin, and TTF-1 were negatively expressed in the present case. Further, Ki67 positivity of <10% in most regions, and of approximately 30% within the hotspots of the glandular ductal region was evident. The results mostly concurred with reports in literature, including negative results for TTF-1 (6, 7, 13). The only discrepancy with reported studies was that the Ki-67 positivity index in our case was lower than that previously reported (15). MLA is known to harbor unique genomic alterations, including, high-frequency KRAS gene mutations and increased chromosome 1q numbers (4, 5, 18), with a mutation rate of 12/16 (75%) in the KRAS gene (19). Molecular detection (Next-generation sequencing method): No clinically significant variants detected. Despite negative molecular test results, the tumor in this case was positive for all the three immuno-histochemical markers, including, PAX-8, CD10, and GATA-3 that effectively diagnose tumors of mesonephric ductal remnants and Müllerian ductal origin (10, 20–27).

In view of the clinical features, histological findings, and immuno-histochemical results, a pathological diagnosis of retroperitoneal MLA was established. Slight discrepancies between our results and those in literature on MLA in the female genitalia were evident in the context of the relatively low mitotic count and Ki67 positivity index in our case. Additionally, the patient did not undergo any postoperative treatment and had a good prognosis at the last follow-up, which categories his tumor as a low-grade malignant cancer. This is in marked contrast to the aggressive clinical course of MLA in females that has a high recurrence rate and distant metastasis (1, 9, 13, 28–30). MLAs may therefore not be exclusively associated (1) with the patient’s gender, (2) tumor site, (3) known molecular associations, and specific pathogenesis. (4) The justification of categorizing of a few well-moderately differentiated malignant tumors as MLA is thus an open question. Given that these tumors are rare, and this is the first case involving a male patient with an MLA located in the retroperitoneum, several more data points are essential to reach a conclusive consensus.

The low incidence, diverse histological patterns, and lack of specific ultrastructural features, make the pathological diagnosis of MLA difficult. A rare occurrence in a male at a specific site, as in our case, made the diagnosis even more challenging. MLAs are included in the differential diagnoses of a multitude of clinical lesions, and patho-morphology, immuno-histochemistry, and molecular testing results in tandem aid its distinction from other tumors, including, mesangial tubular remnants and hyperplasia, malignant mesothelioma, plasmacytoid carcinoma, clear cell carcinoma, and mesenchymal stromal tumors.

To summarize, MLA are rare and can occur in men. It is often misdiagnosed by clinico-pathologists owing to the lack of specific clinical manifestations and diverse pathological patterns. Conclusive diagnosis therefore mandates extensive sampling, careful observation of histological images, and a keen search for characteristic clues, in combination with immuno-histochemical and molecular tests and other auxiliary assessments. MLAs in women are described as a morphologically highly differentiated and aggressive malignancy, that require extensive treatment and adjuvant therapy, including radiotherapy and/or chemotherapy post-surgical resection in early stage patients. Further, patients with MLA harboring KRAS/NRAS gene mutations may be treated with RAS gene-targeted inhibitors. However, currently consensus recommendations for treatment regimens are lacking. Herein, we have brought forth for the first time a case of a male MLA patient, and consequently propose the concept of low-grade malignant MLA that is in sharp contrast to the low-intermediate grade female MLA tumors. Further validation of this concept via long-term follow-up and analysis of a larger number of cases is essential.

## Data Availability

The datasets presented in this study can be found in online repositories. The names of the repository/repositories and accession number(s) can be found in the article/supplementary material.
